# Neuroplasticity‐Driven Mechanisms and Therapeutic Targets in the Anterior Cingulate Cortex in Neuropathic Pain

**DOI:** 10.1002/brb3.71408

**Published:** 2026-04-14

**Authors:** Haibing Xiong, Letai Li, Yanlin Li, Yutong Chen, Jiajie Leng, Tingting Chen

**Affiliations:** ^1^ Department of Neurosurgery Banan Hospital Affiliated to Chongqing Medical University Chongqing China; ^2^ Chongqing Medical University Chongqing China; ^3^ Department of Cardiothoracic Surgery the First Affiliated Hospital of Chongqing Medical University Chongqing China; ^4^ Department of Anatomy and Laboratory of Neuroscience and Tissue Engineering Basic Medical College of Chongqing Medical University Chongqing China; ^5^ School of Nursing Chongqing Medical University Chongqing China; ^6^ Shanghai Jiao Tong University School of Nursing Shanghai China

**Keywords:** anterior cingulate cortex (ACC), circuit remodeling, neuropathic pain, neuroplasticity

## Abstract

**Background:**

Neuropathic pain is a chronic condition initiated by nerve injury and frequently accompanied by affective disturbances, including anxiety and depression. Growing evidence suggests that maladaptive neuroplasticity in the anterior cingulate cortex (ACC) contributes to the persistence and affective dimension of neuropathic pain.

**Objective:**

To narratively review and critically synthesize current evidence on ACC‐related neuroplasticity in neuropathic pain across molecular, circuit, glial, and translational domains.

**Methods:**

We narratively reviewed experimental and clinical studies addressing ACC‐related molecular signaling, synaptic and circuit remodeling, glial and neuroimmune mechanisms, and interventional approaches relevant to neuropathic pain and its affective dimension.

**Results:**

At the molecular level, abnormal ACC synaptic plasticity has been associated with long‐term potentiation involving N‐methyl‐D‐aspartate (NMDA) receptors—particularly GluN2B‐dependent signaling—while the brain‐derived neurotrophic factor (BDNF)–TrkB axis may further contribute to dendritic remodeling and maladaptive synaptic strengthening. At the circuit level, the ACC interacts with limbic regions including the insula and amygdala, within distributed networks that appear to contribute to aversive learning and pain‐related affect. At the non‐neuronal level, alterations in the ACC microenvironment include astrocyte‐linked neuroinflammation and microglia‐associated synaptic remodeling, which may shift excitation–inhibition balance. Therapeutically, ACC‐targeted strategies are evolving from broad pharmacological modulation toward more spatially specific neuromodulation, although major translational challenges remain, including limited target specificity, cross‐species differences, and uncertain causal inference in humans.

**Conclusions:**

ACC‐related neuroplasticity appears to be an important component of neuropathic pain–affect pathophysiology. Future progress will depend on integrating mechanistic insights with network‐level interpretation and improving the precision and clinical translatability of ACC‐engaging interventions.

## Graphical abstract

The anterior cingulate cortex (ACC), as the center for pain and emotion integration, causes integration disorders due to excessive synaptic strengthening and glial cell inflammation/synaptic pruning dysregulation. However, traditional drugs have insufficient targeting, and closed‐loop DBS improves symptoms by precisely regulating ACC.

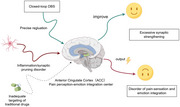

## 1 Introduction

Neuropathic pain, defined by the International Association for the Study of Pain (IASP) as chronic pain arising from lesions or diseases affecting the somatosensory nervous system (Scholz et al. 2019; McIlwrath et al. 2020), affects approximately 10% of the global population with characteristic comorbidities including anxiety, depression, and catastrophizing (Scholz et al. 2019). Unlike acute nociceptive pain, this condition exhibits chronicity, often persisting beyond tissue healing, and frequently manifests as treatment‐resistant recurrent episodes (Guntel et al. 2021; Bouchenaki et al. 2019). The clinical challenge is compounded by a bidirectional relationship between chronic neuropathic pain and emotional disturbances—sleep disturbances and affective disorders not only reduce quality of life but may exacerbate central sensitization processes (McIlwrath et al. 2020; Guntel et al. 2021). These non‐sensory symptoms substantially shape disease burden and may contribute to pain persistence, yet they are often insufficiently addressed by current first‐line pharmacological treatments. This therapeutic gap has prompted increasing interest in central mechanisms that link pain processing with affective and cognitive dysfunction (Bouchenaki et al. 2019).

Among the cortical regions implicated in chronic pain, the anterior cingulate cortex (ACC) has emerged as an important hub within pain‐related affective and salience‐processing networks (Acuña et al. 2023; Jensen et al. 2016). Human neuroimaging studies have reported increased activity in the dorsal ACC in patients with neuropathic pain, while preclinical studies suggest that activation of ACC‐related pathways can lower the threshold for injury perception and promote anxiety‐like or affective behaviors (Jensen et al. 2016, X. Li et al. 2023). However, the ACC should not be interpreted as an isolated generator of chronic pain. Rather, its contribution is better understood within distributed cortical–subcortical systems involved in nociception, emotional appraisal, salience attribution, and behavioral adaptation. (Acuña et al. 2023, Jensen et al. 2016, X. Li et al. 2023)

The chronicity of neuropathic pain is closely linked to structural and functional remodeling of the ACC, including synaptic transmission abnormalities and altered circuit excitability after nerve injury (Ren et al. 2022; Lee et al. 2021). Microglia‐mediated complement C1q‐dependent synaptic pruning may disrupt inhibitory/excitatory balance, while astrocytic downregulation of the glutamate transporter GLT‐1 can elevate extracellular glutamate and create a hyperexcitable neuronal microenvironment (Wei et al. 2024; de Ceglia et al. 2023). Glial remodeling may further influence negative affect via ACC‐related projection pathways, including circuits involving the amygdala (Xiao and Zhang 2018). At the translational level, closed‐circuit deep brain stimulation (DBS) targeting ACC circuits has emerged as a promising approach for refractory neuropathic pain, and optogenetic studies suggest that selective engagement of ACC projections may improve pain‐related cognitive dysfunction (X. Li et al. 2023; Boccard et al. 2017; Leplus et al. 2022).

This review summarizes current evidence on how neuroplastic changes within the ACC contribute to the affective–motivational dimension of neuropathic pain, while recognizing that pain‐related behaviors emerge from distributed cortical–subcortical networks. We integrate molecular, circuit, and glial mechanisms, and we discuss both pharmacological and neuromodulatory strategies that may engage ACC‐related circuits. We also highlight emerging modulators such as sex and circadian influences, and outline key controversies, evidence gaps, and translational challenges.

## 2 Mechanisms of Neuroplastic Remodeling in the Anterior Cingulate Gyrus

The chronicity of neuropathic pain is closely linked to structural and functional remodeling of the ACC. This remodeling involves multilevel plasticity changes at the molecular, cellular, and circuit levels, ranging from synaptic transmission abnormalities and remodeling of dendritic morphology to reorganization of networks across brain regions, and provides the pathological basis for the persistent amplification of the affective dimension of pain.

### 2.1 N‐Methyl‐D‐Aspartate (NMDA) Receptor‐Gated Metaplasticity in ACC Circuits

Central synapses exhibit synaptic plasticity as long‐term potentiation (LTP) and long‐term depression (LTD). Metaplasticity refers to activity‐dependent changes in the thresholds and rules for inducing LTP/LTD, thereby shaping when potentiation versus depression will occur. Although AMPA (α‐amino‐3‐hydroxy‐5‐methyl‐4‐isoxazolepropionic acid) receptors mediate the majority of fast excitatory neurotransmission, NMDA receptor activation is crucial for regulating the induction of bidirectional synaptic plasticity. This functional dichotomy establishes NMDA receptors as molecular gatekeepers of synaptic efficacy adjustments, despite their limited contribution to baseline synaptic transmission under resting membrane potentials (Zhuo 2024).

As a predominant ionotropic glutamate receptor subtype in the mammalian brain, the NMDA receptor adopts a heterotetrameric configuration composed of two obligatory GluN1 subunits paired with either two GluN2A‐D regulatory subunits. Notably, in corticothalamic circuits encompassing the ACC, GluN2A and GluN2B emerge as the predominant GluN2 isoforms, exhibiting distinct developmental trajectories and pharmacological profiles that collectively govern activity‐dependent synaptic plasticity mechanisms (Franchini et al. 2020). Their differential expression patterns (GluN2B predominance in early development vs. GluN2A upregulation in mature synapses) and calcium permeability modulations position these subunits as critical determinants of dendritic integration rules (Amin et al. 2023). It has been shown that GluN2A‐ and GluN2B‐containing NMDARs contribute to cortical plasticity in a context‐dependent manner; in neuropathic pain models, evidence often implicates enhanced GluN2B‐dependent signaling in maladaptive potentiation, but subunit roles should not be treated as intrinsically “inhibitory” or “excitatory” without specifying the synapse/circuit and readout (Xue et al. 2021). This suggests that GluN2B is a mechanistically informative target, although its therapeutic translation remains challenging (Zhuo 2024). Beyond its role in neuropathic pain, the GluN2B subunit is also prominently implicated in the pathophysiology of major depressive disorder (MDD). Preclinical studies link heightened GluN2B function in limbic regions to synaptic and behavioral deficits in depression models (K. Zhang et al. 2021). This evidence has spurred clinical trials of selective GluN2B antagonists for treatment‐resistant depression, though their development faces challenges, including limited efficacy and side effects (Wang, Wang et al. 2021). However, within the specific context of ACC‐mediated neuropathic pain, targeting GluN2B aims to reverse maladaptive plasticity underlying the affective dimension of pain. Key challenges for therapeutic translation include poor blood–brain barrier penetration, dissociative side effects (shared with broad NMDA receptor antagonists), and, critically, the lack of technologies for ACC‐specific drug delivery. While shared GluN2B dysregulation likely contributes to the frequent comorbidity between NP and depression, precisely modulating ACC GluN2B circuits remains paramount for addressing the affective burden of NP (Egunlusi and Joubert 2024; Sherwani et al. 2022).

### 2.2 Brain‐Derived Neurotrophic Factor (BDNF)‐TrkB Signaling Drives Dendritic Remodeling

**FIGURE 1:**
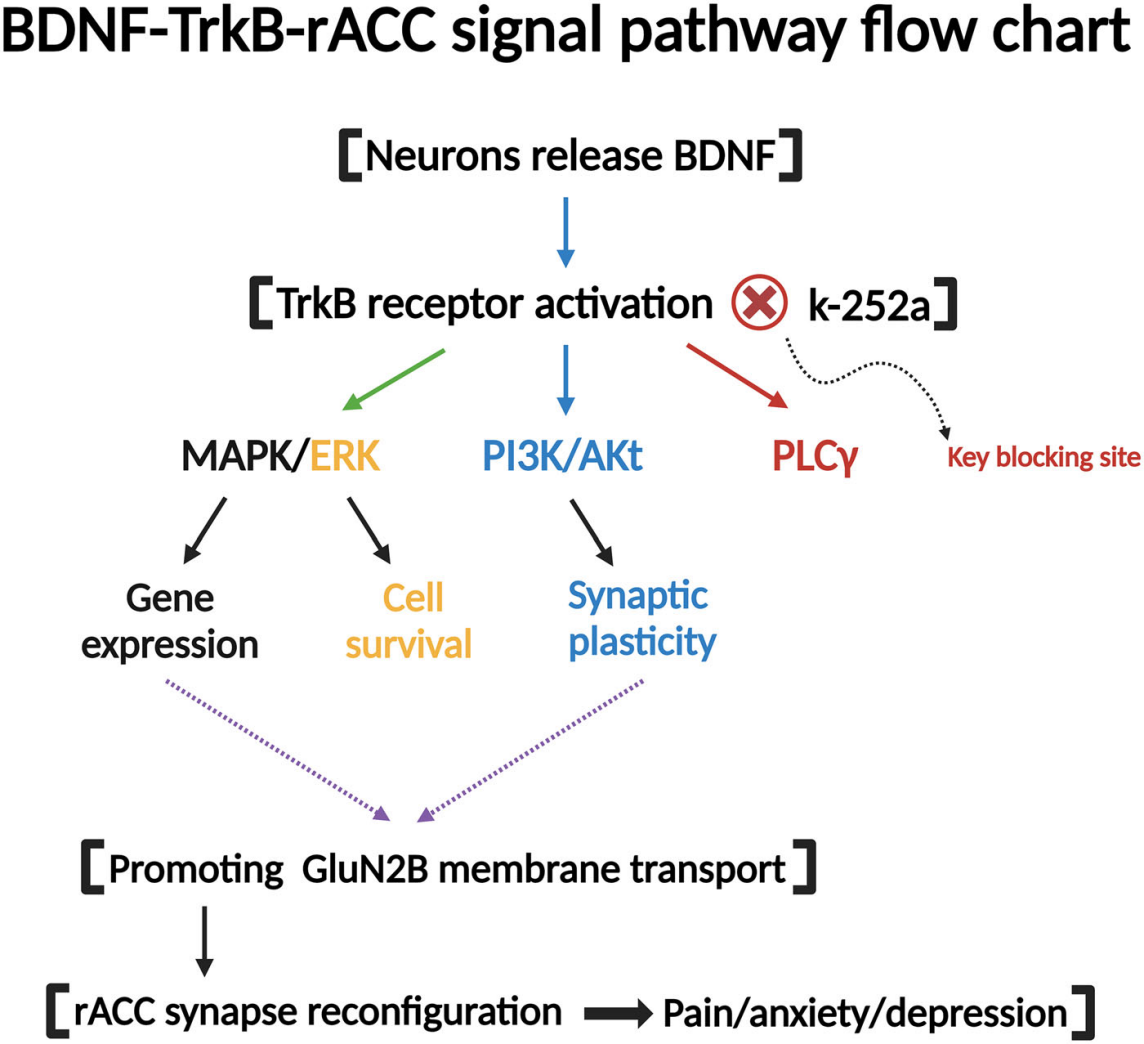
BDNF‐TrkB‐rACC signal pathway flow chart. Solid arrows indicate experimentally supported signaling relationships in preclinical studies; dashed arrows indicate proposed or indirect interactions with limited direct validation in human neuropathic pain. Akt, protein kinase B; BDNF, brain‐derived neurotrophic factor; ERK, extracellular regulated protein kinases; k‐252a, antibiotic k‐252a; MAPK, mitogen‐activated protein kinase; PI3K, phosphatidylinositide 3‐kinase; PLCγ, phospholipase C‐gamma; rACC, rostral anterior cingulate cortex; TrkB, tyrosine kinase receptor B.

The BDNF‐proto‐myosin receptor kinase B (TrkB)‐rostral anterior cingulate cortex (rACC) signaling pathway (for details, refer to Figure 1) plays a significant role in the regulation of emotions associated with neuropathic pain (P. Yang et al. 2023).

BDNF, a neurotrophic factor that has been extensively studied and well‐characterized in the central nervous system, regulates numerous cellular processes involved in the development and maintenance of normal brain function by binding and activating TrkB, a member of the large family of Trk receptors (Numakawa et al. 2018).

BDNF plays a central regulatory role in dendritic remodeling by activating its high‐affinity TrkB. BDNF released from neurons binds to TrkB and triggers receptor dimerization and autophosphorylation, which in turn activate multiple downstream signaling pathways to synergistically regulate the dynamic changes in dendritic structure and function (Huang et al. 2020). The mitogen‐activated protein kinase/extracellular signal‐regulated kinase (MAPK/ERK) pathway promotes the synthesis of synapse‐associated proteins through the activation of transcription factors and drives the extension and stabilization of dendritic arborization, the phosphatidylinositol 3‐kinase/protein kinase B (PI3K/Akt) pathway maintains the integrity of the dendritic network by inhibiting apoptotic signaling and prevents degenerative changes, and the phospholipase C γ (PLCγ) pathway mediates calcium release and protein kinase C (PKC) activation by hydrolyzing phosphatidylinositol 4,5‐bisphosphate (PIP2, it is a substrate for the phospholipase PLCγ, which is hydrolyzed to produce IP3 and DAG, initiating downstream signaling) to generate inositol 1,4,5‐trisphosphate (IP3, acts as a second messenger and binds to IP3 receptors on the endoplasmic reticulum, triggering the release of Ca^2^^+^ into the cytoplasm to regulate cell contraction, secretion, gene expression, etc.) and diacylglycerol (DAG, co‐activates PKC with Ca^2^^+^ to regulate cell proliferation, differentiation, and apoptosis). PKC activation dynamically regulates the morphological plasticity and synaptic transmission efficiency of dendritic spines (Jang et al. 2019, X. Xu et al. 2019; Cappoli et al. 2020). The synergistic action of these pathways ultimately leads to LTP and increased dendritic spine density, supporting the neural basis of learned memory. Notably, BDNF‐TrkB signaling also enhances postsynaptic currents and further strengthens functional dendritic connectivity by facilitating synaptic membrane trafficking of the GluN2B subunit of the NMDA receptor (W. Chen et al. 2014). However, aberrant activation of the pathway (e.g., imbalance of synaptic remodeling in the rACC region) can lead to pathological dendritic remodeling, manifesting as pain sensitization and mood disorders. In a preclinical brachial plexus avulsion model, pharmacological interference with TrkB signaling was associated with reduced pain‐related negative affect, supporting the involvement of the BDNF–TrkB axis in maladaptive ACC‐related plasticity (Zhao et al. 2022). However, compounds such as K‐252a are not clinically viable because of poor specificity, off‐target kinase inhibition, and toxicity. Accordingly, these findings should be interpreted as mechanistic proof‐of‐principle rather than as directly translatable therapeutic strategies (Kowiański et al. 2018).

### 2.3 Dynamic Reorganization of the ACC‐Insula‐Amygdala Circuit

**FIGURE 2:**
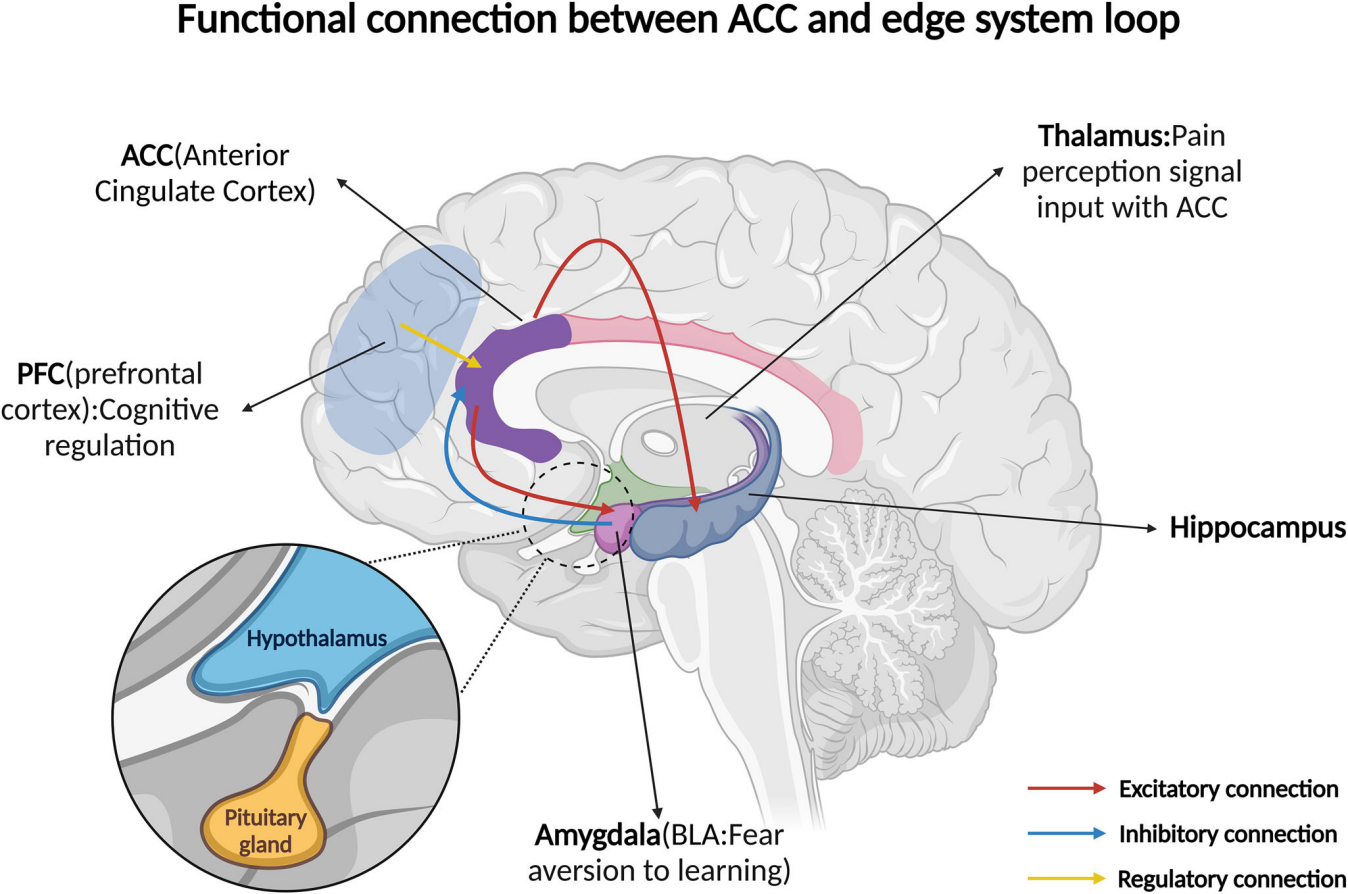
Functional connection between ACC and limbic network. Solid lines indicate experimentally supported preclinical circuit relationships; dashed lines indicate conceptual or inferred network‐level interactions. Human evidence for these circuit mechanisms remains predominantly correlational. ACC, anterior cingulate cortex; BLA, basolateral amygdala; PFC, prefrontal cortex.

The ACC does not operate in isolation (for details, refer to Figure 2), and its pain‐related plasticity is embedded in a broader limbic‐cortical network. Electrophysiological, combined with optogenetic recordings, revealed inhibitory projections between the posterior insula (PIC) and posterior ACC (PACC) that selectively modulate the affective aspects of neuropathic pain (Alonso‐Matielo et al. 2023; Jarrin et al. 2020). The idea that inhibitory pathways between the PIC and PACC can modulate the emotionally motivated aspects of pain highlights these regions as potential targets for the treatment of neuropathic pain. Further network analysis suggests that direct projections from the ACC to the basolateral amygdala (BLA) play a key role in pain aversion learning: chemogenetic inhibition of neurons in the ACC‐BLA reduced conditioned avoidance responses to noxious stimuli in mice, without affecting sensory thresholds (Perumal and Sah 2021). Shen et al. (2025) used astrocyte‐specific iβARK technology and AAV‐shRNA intervention in a chronic neuropathic pain model to target and inhibit the mGluR5/Gq pathway of astrocytes in the ACC region and showed that the mechanical pain threshold was increased in the mGluR5 knockdown group, while microdialysis assays revealed a significant decrease in extracellular glutamate concentration in the ACC region. Ex vivo electrophysiological recordings showed that the intervention reduced the frequency and amplitude of mEPSCs at the ACC‐amygdala synapse, and chemical genetics modulation experiments further confirmed that astrocyte Gq‐DREADD activation specifically enhanced the synaptic transmission efficiency of the ACC‐insular circuits and exacerbated nociceptive hyperalgesia in the CCI model mice. Immunofluorescence quantification showed that astrocyte mGluR5 expression was decreased in the intervention group compared to the control group (Shen et al. 2025). This study suggests that astrocyte mGluR5‐dependent mechanisms may contribute to emotional–cognitive comorbidities of chronic pain by modulating glutamatergic plasticity within ACC‐related circuits. However, the extent to which these findings generalize across pain models, sexes, and species remains uncertain.

### 2.4 Additional Mechanisms Relevant to ACC Neuroplasticity

Autophagy–lysosome dysfunction has recently been linked to persistent pain‐related plasticity in the ACC. In a murine model of chronic postoperative pain, reduced lysosomal hydrolase levels were accompanied by deficiency of the lysosomal trafficking protein TMEM251 (also known as LYSET) in the ACC. TMEM251 overexpression restored autophagic flux, reduced accumulation of synaptic proteins within autophagy substrates, and alleviated pain phenotypes, whereas TMEM251 knockdown was sufficient to induce autophagy impairment and chronic pain‐like behaviors in naive animals (Y. Xu et al. 2025). These findings suggest that impaired proteostasis within ACC neurons can be a disease‐relevant driver of maladaptive synaptic remodeling.

Beyond glutamatergic receptor signaling, second‐messenger pathways that couple activity to excitability changes also contribute to ACC plasticity. A recent study identified cGMP‐dependent protein kinase I (PKG‐I) in the ACC as a facilitator of chronic pain and pain‐associated anxiety/depression, in part by enhancing calcium influx and synaptic potentiation via TRPC channel phosphorylation (T. Wang et al. 2023). In parallel, peripheral nerve injury can engage presynaptic PKG‐I mechanisms in nociceptors that promote cingulate synaptic potentiation through BDNF‐related signaling (Wang, Ma et al. 2021). Together, these data support the NO/cGMP/PKG axis as an additional molecular lever for ACC hyperexcitability in chronic pain.

Conditioning with nociceptive stimuli provides a behavioral framework that bridges sensory inputs and affective learning. The ACC is consistently implicated in aversive learning and conditioned pain‐related avoidance, with manipulations of ACC activity reducing aversion/avoidance even when sensory thresholds are relatively preserved (Kung et al. 2003, Q. Zhang et al. 2017). Incorporating conditioning paradigms therefore helps interpret ACC plasticity as a substrate for pain–emotion memory and maladaptive threat learning, which may be particularly relevant to disability and comorbid anxiety.

We summarize the comparative evidence from animal and human studies on ACC neuroplasticity in neuropathic pain in Table 1.

**TABLE 1:** Comparative evidence from animal and human studies on ACC neuroplasticity in neuropathic pain. Key molecular, circuit, glial, and therapeutic findings are summarized, highlighting translational challenges.

Domain	Species	Key Findings	Supporting references
Molecular mechanisms			
GluN2B‐NMDA LTP	Rodent	GluN2B‐containing NMDA receptor‐dependent LTP in ACC contributes to enhanced excitatory transmission and pain‐related aversion; inhibition reduces aversion/negative affect in multiple models.	(Zhuo 2024; Xue et al. 2021; Egunlusi and Joubert 2024)
	Human	Imaging and pharmacological studies implicate glutamatergic/NMDA mechanisms in affective pain processing, but ACC‐specific GluN2B causal evidence remains indirect in humans.	(K. Zhang et al. 2021; Wang et al. 2021)
BDNF‐TrkB signaling	Rodent	BDNF–TrkB signaling promotes dendritic remodeling and excitatory synaptic potentiation in ACC; blocking TrkB signaling reduces pain‐related negative affect in preclinical models.	(P. Yang et al. 2023; Huang et al. 2020; Zhao et al. 2022)
	Human	No direct ACC causal data; BDNF‐related variation and peripheral BDNF changes correlate with chronic pain susceptibility (indirect evidence).	(Numakawa et al. 2018; Cappoli et al. 2020)
cGMP–PKG‐I signaling	Rodent	PKG‐I in ACC facilitates neuronal hyperexcitability and synaptic potentiation (e.g., via TRPC channels); peripheral injury can engage presynaptic PKG‐I mechanisms that promote cingulate potentiation.	(L. Zhang et al. 2011; J. Li et al. 2020)
	Human	Direct ACC PKG‐I evidence is limited; translational biomarkers for the NO/cGMP/PKG axis in ACC remain to be established.	
TMEM251/LYSET‐dependent autophagy	Rodent	TMEM251/LYSET deficiency in ACC disrupts lysosome trafficking and autophagy, promotes accumulation of synaptic proteins, and contributes to chronic postoperative pain phenotypes.	(Inoue and Tsuda 2018)
	Human	No direct ACC human evidence; autophagy–lysosome dysfunction is a plausible contributor requiring translational validation.	
Neural circuits			
ACC‐insula‐amygdala	Rodent	ACC interactions with insula and amygdala shape aversion learning and affective responses; circuit‐specific manipulations can reduce pain‐related avoidance while sparing baseline sensory processing.	(Alonso‐Matielo et al. 2023; Perumal and Sah 2021; Shen et al. 2025)
	Human	Connectivity changes among ACC, insula, and limbic regions correlate with pain affect and disability, but causal directionality remains uncertain.	(Scholz et al. 2019; McIlwrath et al. 2020)
Glial mechanisms			
Astrocyte inflammation	Rodent	Pro‐inflammatory astrocytic transformation (e.g., impaired glutamate homeostasis) contributes to ACC hyperexcitability and pain–anxiety comorbidity; astrocyte modulation can reduce hypersensitivity and negative affect.	(Wei et al. 2024; Q. Zhou et al. 2025)
Microglia pruning	Rodent	Microglia‐dependent synaptic pruning imbalance (e.g., CXCL12/CXCR4 signaling) may reduce inhibitory inputs and shift the E/I balance toward excitation, potentially contributing to aberrant ACC LTP.	(Gu et al. 2016; Chipman et al. 2022)
Microglia P2×4R–BDNF–TrkB signaling	Rodent	Microglial P2×4R activation promotes BDNF release and neuronal TrkB signaling, enhancing ACC excitability and synaptic plasticity; pathway disruption alleviates pain behaviors in preclinical models.	(Liang et al. 2025)
Interventions			
Pharmacological	Rodent	Systemic analgesics and neuromodulators affect ACC‐related processes but lack spatial specificity; circuit‐relevant targets (e.g., oxytocinergic modulation, and ketamine) show promise in preclinical work.	(Bouchenaki et al. 2019; Akazawa et al. 2019; H. Zhou et al. 2018)
	Human	Clinical pharmacology improves symptoms in subsets but often does not specifically normalize ACC hyperactivity; evidence varies by drug class and outcome domain (sensory vs. affective).	(Bouchenaki et al. 2019; Akazawa et al. 2019; N. Li et al. 2010)
CGRP monoclonal antibodies (indirect ACC evidence)	Human	In migraine, anti‐CGRP monoclonal antibodies can alter brain network activity/connectivity and may be associated with structural or functional changes in pain‐processing cortices; relevance to neuropathic pain affect and ACC mechanisms remains uncertain.	(Basedau et al. 2022; Filippi et al. 2023)
Neuromodulation	Rodent	Circuit‐level neuromodulation (e.g., optogenetic/chemogenetic approaches) demonstrates that ACC subcircuits can modulate aversion, anxiety, and pain‐related decision‐making.	(Alonso‐Matielo et al. 2023; Jarrin et al. 2020; Hansson et al. 2021; Burke et al. 2019)
	Human	rTMS/iTBS and DBS approaches can modulate pain‐related networks, but controlled evidence for ACC‐targeted efficacy and parameter optimization remains limited.	(Leplus et al. 2022; Hansson et al. 2021; Vogt and Paxinos 2014)

## 3 Glial Cell‐Mediated Imbalance in the ACC Immune Microenvironment

**FIGURE 3:**
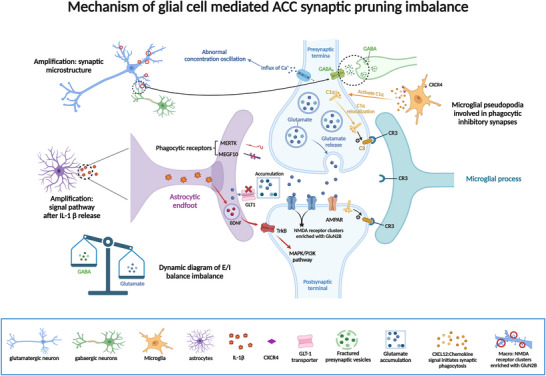
Mechanism of glial cell mediated ACC synaptic pruning imbalance. Solid arrows indicate experimentally supported mechanisms in preclinical studies; dashed arrows indicate proposed or indirect interactions with limited direct validation in human neuropathic pain. ACC, anterior cingulate cortex; AMPAR, a‐amino‐3‐hydroxy‐5‐methyl‐4‐isoxazolepropionic acid receptor; BDNF, brain‐derived neurotrophic factor; C1q, complement component 1q subcomponent; C3, complement 3; CR3, complement receptor 3; CXCL12, chemokine CXC subfamily ligand 12; CXCR4, CXC chemokine receptor 4; E/I, excitatory/inhibitory; GABA, γ‐aminobutyric acid; GLT‐1, glutamate transporter 1 (EAAT2); MAPK, mitogen‐activated‐protein kinase; MEGF10, multiple EGF like domains 10; MERTK, C‐Mer proto‐oncogene tyrosine kinase; NMDA, N‐methyl‐D‐aspartate; PI3K, phosphatidylinositide 3‐kinase; TrkB, tyrosine kinase receptor B.

In addition to neuronal plasticity, glial–neuronal interactions contribute to maladaptive ACC microenvironmental remodeling in neuropathic pain (Wang et al. 2021; T. Wang et al. 2023; König et al. 2021; Piao et al. 2018). Activated astrocytes and microglia may amplify cortical hyperexcitability through the release of pro‐inflammatory mediators, altered glutamate homeostasis, and dysregulated synaptic remodeling (Wei et al. 2024; de Ceglia et al. 2023; Cserép et al. 2020, König et al. 2021; Piao et al. 2018). These processes may link neuroimmune signaling to the affective burden of chronic pain by disturbing local synaptic balance within ACC‐related circuits (Z. Chen et al. 2019; Brifault et al. 2019, Y. Zhang et al. 2022). In summary, ACC astrocytes and microglia together shape immune microenvironmental imbalance in pain‐related signaling by releasing pro‐inflammatory mediators and modulating synaptic homeostasis (for details, see Figure 3).

In addition to chemokine‐driven pruning pathways, purinergic microglia–neuron communication has emerged as another mechanism by which glia can tune ACC excitability. In a recent study of muscle pain chronicity, activation of microglial P2×4 receptors in the ACC promoted BDNF release and downstream neuronal TrkB signaling, enhancing synaptic plasticity and cortical hyperexcitability; disrupting P2×4R–BDNF–TrkB signaling reduced neuronal excitability and alleviated pain behaviors (Liang et al. 2025). While pain etiologies differ across models, these data support the broader concept that microglial purinergic signaling can amplify ACC plasticity and may represent a pharmacologically tractable node.

Finally, glial and neuronal proteostasis pathways may intersect with synaptic remodeling. As discussed above, TMEM251/LYSET‐dependent lysosomal trafficking in the ACC regulates autophagy and synaptic protein turnover, and its disruption can promote persistent pain phenotypes (Y. Xu et al. 2025). Whether microglia‐dependent pruning and autophagy dysfunction converge on shared synaptic substrates in the ACC remains an open question.

### 3.1 Pro‐Inflammatory Transformation of Astrocytes

Astrocytic inflammatory transformation has been implicated in chronic pain‐related cortical dysfunction across several brain regions (Guida et al. 2020; Ma et al. 2021). In the ACC, pro‐inflammatory astrocytic activation may disrupt local excitatory/inhibitory balance through the release of inflammatory mediators such as TNF‐α and IL‐6 together with impaired glutamate reuptake (Wei et al. 2024; de Ceglia et al. 2023, Q. Zhou et al. 2025). These changes may promote cortical hyperexcitability and contribute to the co‐occurrence of neuropathic pain and anxiety‐like behaviors (Wei et al. 2024, Q. Zhou et al. 2025).

### 3.2 Microglia‐Dependent Dysregulation of Synaptic Pruning

**FIGURE 4:**
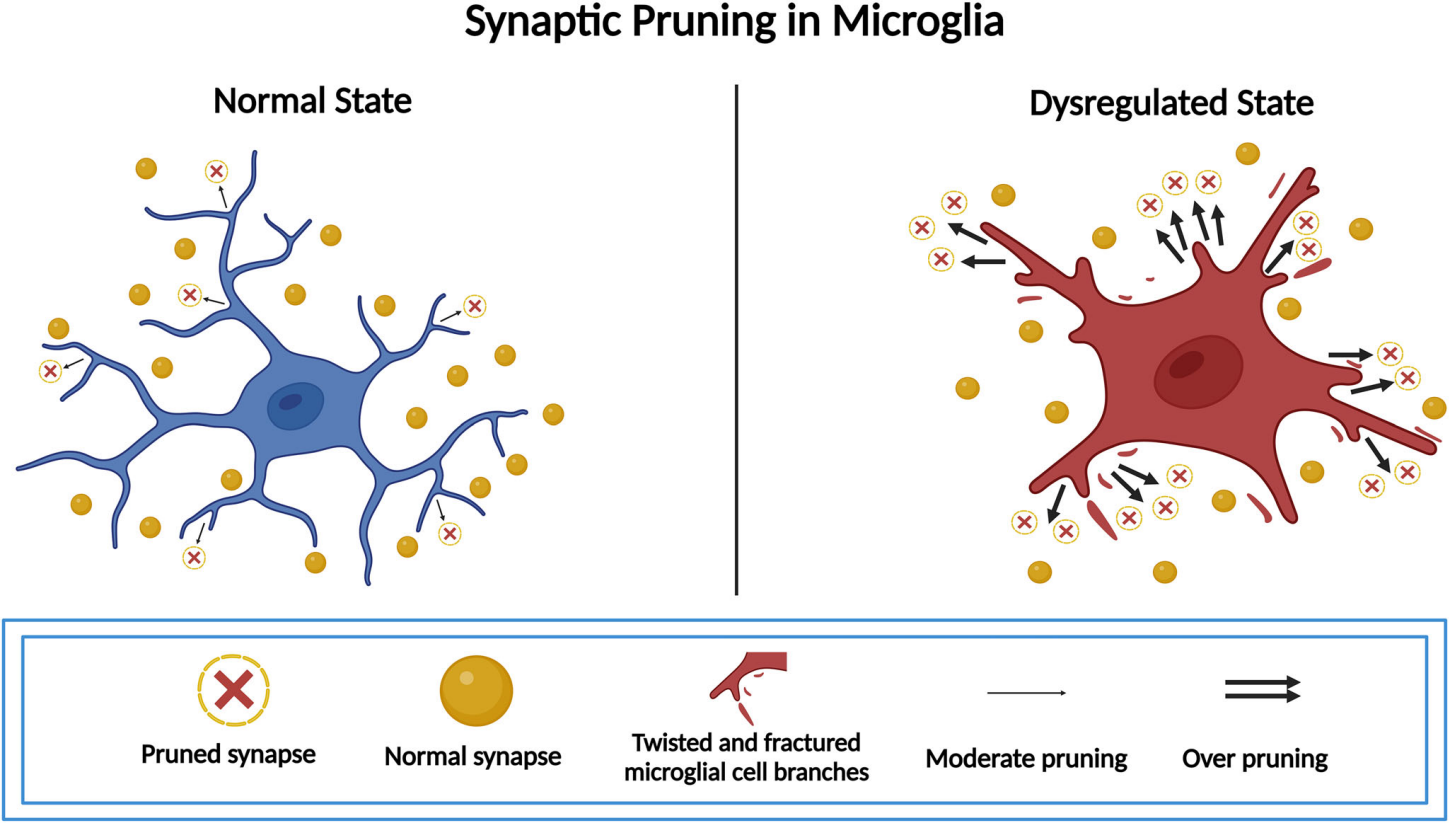
Synaptic pruning in microglia. Solid arrows indicate experimentally supported mechanisms in preclinical studies; dashed arrows indicate proposed or indirect interactions with limited direct validation in human neuropathic pain.

Microglia serve as crucial modulators of neuronal connectivity, excitability, and synaptic plasticity (Cserép et al. 2020, Badimon et al. 2020). In neuropathic pain, ACC pathological remodeling involves not only intrinsic neuronal plasticity alterations but also significant dysregulation of microglia‐mediated synaptic pruning homeostasis(For details, refer to Figure 4). Experimental evidence reveals that nerve injury triggers significant synaptic phagocytic phenotypic remodeling of ACC microglia (Gu et al. 2016). The specific CXC chemokine ligand 12 (CXCL12)/CXC chemokine receptor 4 (CXCR4) signaling pathway was confirmed to be specifically activated in such microglial cells using 3D imaging and multimodal electrophysiological techniques (Song et al. 2023). CXCL12 is a core member of the chemokine family that binds to the CXCR4 receptor to dual regulate immune cell migration (e.g., directed recruitment of microglia to the ACC injury zone) and remodeling of the synaptic microenvironment (induction of AMPA receptor endocytosis and enhancement of LTD) (Zweemer et al. 2014). CXCR4 is a member of the CXC chemokine receptor family and belongs to the G protein‐coupled receptor (GPCR) superfamily, whose core function is to mediate chemokine signaling and regulate cell migration, survival, and inflammatory responses (Cheng et al. 2023; Kawaguchi et al. 2019). CXCL12 induces abnormal phagocytosis of inhibitory γ‐aminobutyric acid (GABA)‐ergic synapses, leading to a decrease in local inhibitory inputs to the ACC; synaptic phagocytosis activity of microglia is biased toward inhibitory circuits, while pruning of glutamatergic excitatory synapses is insufficient, resulting in over‐accumulation of NMDA receptors (especially the GluN2B subunit) in the postsynaptic membrane. The dysregulation of synaptic pruning leads to a relative enhancement of excitatory inputs (increase in glutamate release) and an absolute attenuation of inhibitory inputs (GABA release). The absolute attenuation (reduced GABA release) significantly enhances the intrinsic excitability of ACC pyramidal neurons and induces aberrant maintenance of LTP (Luo et al. 2016; Chipman et al. 2022). Interestingly, CXCL12/CXCR4 signaling both directly promotes synaptic phagocytosis in microglia and indirectly increases activity‐dependent BDNF secretion in glutamatergic neurons (Coull et al. 2005). This positive feedback loop further enhances the activity‐dependent remodeling of the ACC network, suggesting that microglial synaptic pruning receptors such as CXCR4 may represent mechanistically informative targets for future investigation, although their therapeutic relevance in humans remains uncertain.

## 4 Intervention Strategies and Clinical Challenges in Targeting ACC

Current ACC‐targeted pain modulation strategies have evolved from conventional pharmacology to precision neuromodulation, but their clinical translation remains limited by therapeutic target heterogeneity, limited spatial resolution, and interspecies mechanistic disparities.

### 4.1 Limitations of Existing Drugs

Pharmacological treatment of neuropathic pain continues to confront the dual insufficient multi‐target modulation and limited spatial specificity. Based on the principle of step therapy, commonly used drugs are categorized based on efficacy as follows: (1) first‐line: α2δ calcium channel modulators (e.g., gabapentin and pregabalin), norepinephrine (NE)/5‐hydroxytryptamine reuptake inhibitors (SNRIs), and tricyclic antidepressants; (2) second‐line: topical capsaicin patches/lidocaine; and (3) third‐line: tramadol/strength opioids (Bouchenaki et al. 2019; Akazawa et al. 2019). However, the efficacy of these drugs is limited by their inability to precisely target the pathological hyperexcitability of the ACC, an important mechanism relevant to the affective dimension of pain.

At the molecular level, modulation of the ACC by existing drugs represents a dynamic paradox: NE and dopamine (DA) functionally synergize to maintain the E/I input balance, although they regulate the ACC network by antagonizing different receptor subtypes (e.g., α2A versus D1 receptors) in pyramidal neurons (Lançon et al. 2021; Koga et al. 2020). However, pharmacokinetic properties (e.g., blood–brain barrier penetration efficiency) and heterogeneity of receptor distribution significantly reduce their regulatory specificity. Clinical studies suggest that gabapentin and inhaled nitrous oxide can modulate ACC signals in humans, but the direction and mechanistic interpretation depend on the imaging modality, task context, and network state; changes in ACC activity may reflect altered salience/attention or disinhibition rather than direct glutamatergic activation. Restoration of specific subcircuits may be preferable to widespread inhibition of somatosensory signal filtering (Acuña et al. 2023; Weinrich et al. 2025).

Recent studies have revealed a more targeted regulatory strategy: the oxytocin system within the ACC can reduce nociceptive sensitization while ameliorating anxiety‐like behaviors by inhibiting NMDA receptor‐dependent LTP formation in the presynaptic membrane (X. Li et al. 2021; Shi et al. 2024; Crane et al. 2020). Such findings suggest that targeted interventions based on subregions of the ACC network/specific neuromodulatory pathways may overcome the nonselective limitations of conventional drugs.

Ketamine, a NMDA receptor antagonist with rapid‐acting antidepressant effects, represents a notable exception relevant to ACC modulation in both pain and depression (Siegel et al. 2021, H. Zhou et al. 2018). Sub‐anesthetic doses of ketamine produce complex effects, including the acute blockade of NMDA receptors in GABAergic interneurons. This leads to disinhibition and a massive release of glutamate, which subsequently activates AMPA receptors and downstream activation via mammalian target of rapamycin (mTOR)‐dependent synaptic protein synthesis. Notably, modulation of ACC activity and functional connectivity is a pivotal mechanism underlying ketamine's rapid mood‐ and pain‐modifying effects (Suzuki et al. 2017, N. Li et al. 2010; Krystal et al. 2024).

From a clinical perspective, intravenous ketamine has been shown to effectively relieve various chronic pain conditions, such as neuropathic pain, while also improving coexisting depressive symptoms (Orhurhu et al. 2019). This dual effect aligns with its mechanism of action on common neural bases, such as the ACC. However, the use of ketamine for chronic neuropathic pain is limited by several factors. Its effects on pain are often temporary, necessitating repeated administration. It lacks specificity for the GluN2B subunit or the anterior cingulate region. Furthermore, it carries risks of hallucinogenic side effects, dissociative symptoms, hemodynamic changes, and potential for abuse (Cadavid et al. 2023, Rhee et al. 2022). These limitations highlight the need for more targeted approaches, such as GluN2B‐specific modulators or anterior cingulate‐specific neuromodulation, to achieve sustained relief of the emotional dimension of neuropathic pain and enhance safety.

Biologic therapies aimed at calcitonin gene‐related peptide (CGRP) signaling—particularly monoclonal antibodies used for migraine prevention—have prompted questions about whether peripheral CGRP blockade can secondarily reshape central pain networks. Human neuroimaging studies in migraine suggest that anti‐CGRP monoclonal antibodies can alter activity and connectivity in pain‐processing regions, including the insula and other cortical areas, and may be associated with structural or network‐level changes over weeks to months (Basedau et al. 2022; Filippi et al. 2023). Direct evidence for effects on ACC‐driven affective components in neuropathic pain is currently limited; we therefore discuss CGRP biologics as an emerging, largely indirect line of evidence that highlights the need to distinguish peripheral analgesic mechanisms from central network normalization.

### 4.2 Emerging Neuromodulation Technologies

Repetitive transcranial magnetic stimulation (rTMS) is an effective treatment for neuropathic pain, although the ACC‐iTBS protocol may be the best compromise for an easier‐to‐implement rTMS protocol for the treatment of chronic neuropathic pain (Lefaucheur et al. 2020; Mussigmann et al. 2024). Collectively, available studies suggest that ACC‐DBS may preferentially reduce pain‐related suffering and improve quality of life in selected patients with refractory neuropathic pain; however, the evidence base remains limited by small samples, heterogeneous etiologies, and variable targeting parameters (Boccard et al. 2017).

ACC‐DBS is an invasive but adjustable neurosurgical intervention that can be programmed and, if needed, discontinued or explanted. Consistent with the putative role of the ACC in affective–motivational pain processing, available clinical reports suggest that ACC‐DBS may reduce the suffering/aversiveness of pain more than sensory intensity, but the evidence base remains limited (small samples, heterogeneous etiologies, and variable targeting/parameter settings) (Leplus et al. 2022). Accordingly, ACC‐DBS should be interpreted as a promising yet still investigational approach that requires standardized outcome measures, longer follow‐up, and controlled studies to define efficacy and safety.

Beyond pharmacological and device‐based approaches, lifestyle interventions may also shape ACC plasticity. For example, voluntary running has been reported to ameliorate synaptic degeneration and normalize synaptic plasticity in the ACC in chronic pain states, supporting exercise as a low‐risk adjunct strategy to mitigate maladaptive cortical reorganization. However, ACC‐specific mechanistic evidence in neuropathic pain remains limited, and future work should clarify dose–response relationships and circuit specificity (Miyake et al. 2025, Y. Zhou et al. 2022).

### 4.3 Conversion Bottlenecks and Multidimensional Optimization Strategies

Cross‐species phenotypic mismatch: According to the Brodmann partitioning, the rodent ACC occupies areas 24, 25, and 32, whereas the human ACC occupies areas 24, 25, 32, and 33 (van Heukelum et al. 2020). Studies have shown that the cingulate cortex in both species has broadly similar connectivity, but that the ACC in primates has more extensive and complex connectivity with several higher brain areas, especially in humans (van Hout et al. 2024). The connections between the ACC and the hypothalamus are even more unique and require a special class of neurons: calcineurin‐binding pyramidal neurons (von Economo neurons), which are currently thought to be present mainly in the brains of primates and a small number of large, social mammals, but are not found in lower mammals such as rodents, perhaps because the A24c′ region of the human brain covers a large area of the cingulate cortex and the ACC has been shown to follow a broadly similar pattern (Oane et al. 2023). The A24c′ region is covered by a high density of pyramidal neurons, but since rodents do not have a cingulate sulcus, this region of A24c′ does not exist (van Heukelum et al. 2020). These neurons have a fusiform morphology, which allows for rapid transmission of information and thus involvement in rapid decision making and response, and may be associated with complex social cognition and emotional processing, allowing for more precise and effective processing of emotions and control of autonomic movements in humans, who are therefore more emotionally and cognitively complex than rodents (Oane et al. 2023; López‐Ojeda and Hurley 2022). At the same time, due to differences in rodent and human ACC nomenclature, their Cg1 and Cg2 regions do not include the A25 and A32 regions, which would result in the absence of relevant autonomic responses in animal simulation tests; furthermore, both Cg1 and Cg2 span part of A24 and A24′, and unless these two regions are examined separately, the functional differences between A24 and A24′ would be difficult to demonstrate (van Heukelum et al. 2020). The reliance of preclinical studies on mechanical nociceptive hypersensitivity and conditioned position avoidance (CPA) tests means that rodents are unable to fully mimic the subjective human experience of pain (Hansson et al. 2021; Vogt and Paxinos 2014). These differences limit the applicability of animal models to the study of higher cognitive and emotional functions in humans and, in particular, animal model‐based drug screens may not be able to fully predict therapeutic effects on human pain, especially in the development of drugs targeting the affective dimensions of pain.

Spatial resolution limitations: Existing TMS/DBS techniques have difficulty distinguishing between affective (24a/b) and cognitive assessment (24c) subregions within the ACC, and nonspecific activation may induce decision‐making deficits (Burke et al. 2019; Neudorfer et al. 2023).

Temporal dynamic adaptation: The ACC shows plasticity reversal at different stages of pain chronicity—early LTP enhancement coexists with late synaptic loss—but most intervention strategies target only a single time window and lack dynamic tracking (Corder et al. 2019).

In response to the above challenges, the development of cross‐scale monitoring techniques and artificial intelligence–driven personalized modulation models will be the direction for the next generation of pain neuromodulation (Tervo et al. 2021). Meanwhile, the development of nanocarriers targeting the GluN2B subunit of the ACC will also be a new direction to bridge the blood–brain barrier gap (Wadhwa et al. 2024).

## 5 Discussion

### 5.1 Conceptual Considerations and Alternative Interpretations of ACC Plasticity

A major conceptual issue in this field is whether ACC plasticity should be interpreted as a driver of neuropathic pain itself or, more cautiously, as a substrate of pain‐related learning, salience attribution, and affective adaptation. The ACC is widely involved in aversive learning, conflict monitoring, reinforcement, and emotional salience. Accordingly, increased ACC activity in chronic pain may partly reflect persistent threat prediction, avoidance learning, and negative emotional valuation rather than direct amplification of nociceptive input alone. This interpretation is consistent with studies showing that ACC manipulations often alter pain aversion and affective behavior more robustly than sensory thresholds (Alonso‐Matielo et al. 2023; Jarrin et al. 2020; Perumal and Sah 2021; Shen et al. 2025; Yuan et al. 2024).

A second issue is that ACC dysfunction should be understood within distributed pain‐related networks rather than as an isolated mechanism. Chronic pain emerges from interactions among cortical and subcortical regions, including the insula, amygdala, thalamus, prefrontal cortex, hippocampus, and brainstem modulatory systems. Therefore, emphasizing the ACC alone risks oversimplifying the network architecture of pain chronification (Alonso‐Matielo et al. 2023; Jarrin et al. 2020; Perumal and Sah 2021; Shen et al. 2025; Yuan et al. 2024).

A third issue concerns biological specificity. Several mechanisms discussed in this review—including BDNF–TrkB signaling, NMDA receptor‐dependent potentiation, autophagy‐related proteostasis, and microglia‐associated synaptic remodeling—are not unique to neuropathic pain. Rather, they are general mechanisms of cortical plasticity that also operate in learning, stress responses, and emotional regulation (Franchini et al. 2020; Amin et al. 2023; Xue et al. 2021, P. Yang et al. 2023; Numakawa et al. 2018; Huang et al. 2020; Jang et al. 2019, X. Xu et al. 2019; Cappoli et al. 2020, W. Chen et al. 2014, Y. Xu et al. 2025, T. Wang et al. 2023; Wang et al. 2021; Cserép et al. 2020; Badimon et al. 2020).

Despite broad agreement that the ACC is an important node in pain‐related affective processing, substantial conceptual and translational controversies remain.

Given the high burden of affective comorbidities, we also emphasize the potential role of stress‐related neuromodulatory systems. The locus coeruleus–norepinephrine (LC–NE) system innervates the ACC and can regulate arousal, salience attribution, and threat learning—processes that are frequently dysregulated in post‐traumatic stress disorder (PTSD). Preclinical evidence indicates that chronic stress can exacerbate neuropathic pain via ACC‐related pathways, suggesting that NE‐dependent state variables may gate ACC plasticity and influence pain chronification (X. Yang et al. 2025; Sherin and Nemeroff 2011).

Recent studies have revealed a sex dimorphism in the regulation of pain pathways by glial cells. Studies have shown significant sex differences in pain transmission mechanisms and expression of cortical plasticity (Osborne et al. 2021; Stratton et al. 2024; Deal et al. 2022). Clinical data showed that for mean pain intensity in patients with neuropathic pain, the mean NRS score for females was 4.90 ± 1.79, which was significantly higher than that of males (4.37 ± 1.79; *p* = 0.03) (Kim, Lee, Kim et al. 2020). This suggests that there are differences in the expression of pain between males and females. The animal model also suggests that the sensitization to nerve injury in male mice is primarily driven by microglia, whereas the same process in female mice relies on T‐cell mediation, suggesting a sex‐specific pattern of division of labor for neuroimmune‐interacting molecules (Kim, Lee, Kim et al. 2020). In the cortex of healthy mice, there was no sex difference in LTP in the ACC and somatosensory cortex, but male ACC showed stronger LTD. Early/late LTP (e‐LTP/l‐LTP) at hippocampal SC‐CA1 synapses also did not differ between sexes, whereas l‐LTP at TA‐CA1 synapses was more pronounced in males (Qi et al. 2016). The study showed that optogenetic activation of the ACC–ACC pathway in mice of both sexes resulted in significant reductions in mechanical withdrawal thresholds and hot plate response latencies after blue light (465 nm) stimulation in both sexes, but affective behaviors showed sex‐specificity: only males showed a reduction in open‐arm exploration time in the EPM, whereas females did not show significant changes, and there were no differences in locomotor activity and absenteeism test metrics between the two groups (X. Li et al. 2023). The above findings suggest that sex‐specific glial modulation may induce conserved damage in the same cortical circuits through differential synaptic plasticity mechanisms. Therefore, the future work should test whether sex‐dependent CXCR4 signaling differences translate into clinically actionable stratification, while avoiding premature therapeutic claims before robust human evidence may be a new direction for future research.

Circadian disruption is closely associated with clinical symptom fluctuations in chronic pain, but its central regulatory mechanisms have not been elucidated (Kim, Lee, Koike et al. 2020). Recent studies have shown that BMAL1, the microglial core clock gene of the ACC, may be a key target to explain the circadian fluctuations of pain by regulating the rhythmic secretion of neuroinflammatory factors (X. Wang et al. 2020). Experiments showed that glial‐specific BMAL1 knockout mice lost circadian differences in nociceptive sensitivity in an inflammatory pain model, accompanied by disturbed phasic expression of IL‐1β and TNF‐α (Gaudet et al. 2018). Single‐cell sequencing further revealed that BMAL1 modulates ACC microglia‐neuron synaptic pruning rhythms through direct binding to the CX3C chemokine receptor 1 (CX3CR1) promoter region, leading to daytime‐specific enhancement of glutamatergic synapses, suggesting that targeting glial biological clock components may optimize time‐dependent analgesic strategies (Jiao et al. 2024). On this basis, biological clock‐based timed drug delivery strategies may be considered for neuropathic pain patients in the future.

## 6 Conclusions

This review summarizes ACC‐related neuroplasticity relevant to neuropathic pain at the molecular, circuit, and cellular levels. At the molecular level, GluN2B‐containing NMDA receptor signaling and BDNF–TrkB‐associated dendritic remodeling appear to contribute to maladaptive excitatory plasticity in the ACC (Zhuo 2024; Franchini et al. 2020; Amin et al. 2023; Xue et al. 2021, K. Zhang et al. 2021; Wang et al. 2021; Egunlusi and Joubert 2024; Sherwani et al. 2022, P. Yang et al. 2023; Numakawa et al. 2018; Huang et al. 2020; Jang et al. 2019, X. Xu et al. 2019; Cappoli et al. 2020, W. Chen et al. 2014; Zhao et al. 2022; Kowiański et al. 2018). At the circuit level, the ACC interacts with limbic regions such as the insula and amygdala to shape aversive learning and emotional comorbidities (Yuan et al. 2024). At the cellular level, glial mechanisms—including astrocytic glutamate transporter regulation and microglia‐dependent synaptic remodeling—can bias excitation/inhibition balance and sustain maladaptive network activity. Clinically, neuromodulation and pharmacological approaches show promise but remain constrained by limited targeting precision, heterogeneous patient phenotypes, and cross‐species translational gaps. Future progress will benefit from convergent evidence across species, cell‐type‐resolved mapping, and rigorously controlled clinical studies that test causal hypotheses and define who benefits from ACC‐engaging interventions (Merk et al. 2022; Boutet et al. 2021).

## Author Contributions

All authors contributed to editorial changes in the manuscript. All authors read and approved the final manuscript.

## Funding

This research was funded by Chongqing Science and Health Joint Medical Research Program (2021MSXM261).

## Conflicts of Interest

The authors declare no conflicts of interest.

## Ethics Statement

The authors have nothing to report.

## Data Availability

Data sharing is not applicable to this article as no datasets were generated or analyzed during the current study.
